# 

**Published:** 1961-04

**Authors:** 


### *Contents*

| | | |
|---|---|---|
| CAVE RESCUE | *Dr. Oliver C. Lloyd* | 37 |
| SUBARACHNOID HAEMORRHAGE | *H. Lovell Hoffman* | 50 |
| THE UNSTABLE CERVICAL DISC | *D. M. Jones* | 57 |
| THE SKELETON IN THE PIT | *G. Stewart Smith* | 61 |
| CLONORCHIASIS | *Clinical Pathological Conference* | 65 |
| NOTES AND NEWS | | 71 |
| APPOINTMENTS | | 71 |

